# How does the adoption of nutritional plans influence the performance of meat quail during the growth phase in hot environments?

**DOI:** 10.3389/fvets.2024.1469516

**Published:** 2024-12-10

**Authors:** Luiz Arthur dos Anjos Lima, Maria Isabelly Leite Maia, Larissa Kellen da Cunha Morais, Tarsys Noan Silva Veríssimo, José Danrley Cavalcante dos Santos, Adriano Leite da Silva, Nátali Rodrigues dos Santos, Severino Guilherme Caetano Gonçalves dos Santos, José Helder de Andrade Moura, Rannyelle Gomes Souza, José Humberto Vilar da Silva, Fernando Guilherme Perazzo Costa, Lucas Rannier Ribeiro Antonino Carvalho, Edilson Paes Saraiva

**Affiliations:** ^1^Research Group in Bioclimatology, Ethology and Animal Welfare (BioEt), Department of Animal Science, Federal University of Paraiba, Areia, Paraiba, Brazil; ^2^Department of Animal Science, Federal University of Paraiba, Areia, Paraiba, Brazil; ^3^Department of Animal Production, National Institute of Semiarid - INSA, Campina Grande, Paraiba, Brazil; ^4^Department of Animal Science, Federal University of Tocantins, Araguaina, Tocantins, Brazil; ^5^Department of Animal Science, Federal University of Paraiba, Bananeiras, Paraiba, Brazil; ^6^Karolinska Institutet, Department of Physiology & Pharmacology Biomedicum, Stockholm, Sweden

**Keywords:** amino acids, animal nutrition, crude protein, diet, European quail, heat stress

## Abstract

The present study aimed to evaluate the effects of different nutritional plans on meat quails subjected to heat stress. A total of 324 quails male European quails (*Coturnix coturnix coturnix*) were used, with an average initial weight of 121.48 g ± 3.1 g, distributed in a completely randomized design with nine treatments and six repetitions of six birds each. The diets were evaluated from 22 to 42 days of age, according to the following treatments: T1 - Control diet; T2 - Moderate crude protein (CP) reduction, without amino acid supplementation (AA); T3 - Moderate CP reduction with supplementation of methionine (Met) and cystine (Cys); T4 - Moderate CP reduction with Met, Cys, and lysine (Lys) supplementation; T5 - Moderate CP reduction with Met, Cys, Lys, and threonine (Thr) supplementation; T6 - Severe CP reduction, without AA supplementation; T7 - Severe CP reduction with Met and Cys supplementation; T8 - Severe CP reduction with Met, Cys, and Lys supplementation; T9 - Severe CP reduction with Met, Cys, Lys, and Thr supplementation. The room temperature was maintained at 30°C, with relative humidity at 76.42%, and a black globe humidity index (BGHI) of 82.19. No significant (*p* > 0.05) effect of protein reduction or amino acid supplementation was observed on live weight, feed intake, carcass weight, yield, breast, legs, heart, and gizzard. However, significant effects were observed on weight gain (*p* < 0.04), feed conversion (*p* < 0.05), liver weight (*p* < 0.001), and liver yield (*p* < 0.001). In hot environments, crude protein in the diet of meat quails can be reduced from 22 to 17.6%, with adequate methionine supplementation to achieve 0.800% digestible Met + Cys during the growth phase (22–42 days). These nutritional strategies may optimize performance, reduce costs, and provide environmental benefits by decreasing nitrogen excretion. Future research should investigate the interactions between diet, heat stress, and quail performance, focusing on different amino acid combinations and their impacts on bird health and productivity under varied thermal conditions.

## 1 Introduction

Environments where temperatures exceed the thermal comfort zone of birds lead to heat stress, severely compromising the productivity and welfare of quails (1). Due to their high surface-to-volume ratio and lack of sweat glands, quails are particularly susceptible to rising environmental heat, which hinders the efficient dissipation of excess heat (2). Research indicates that heat stress can occur in quails at temperatures above 28°C, triggering significant physiological changes and negatively affecting critical parameters such as feed intake, egg production, and egg quality (3, 4). These effects not only reduce productive efficiency but also pose significant economic challenges for farms, especially in hot climates.

To address these challenges, nutritional strategies have been explored as additional solutions to environmental improvements to mitigate the impacts of heat stress on quails. A key approach is to reduce dietary heat increment by lowering crude protein levels and incorporating industrial amino acids, which more precisely meet the birds’ protein requirements and avoid excess protein that contributes to additional metabolic heat. This strategy is especially relevant in non-ruminant diets, where protein is a major factor in increasing internal heat, particularly in formulations based on corn and soy, which are commonly used in Brazil (5, 6).

By reducing the inclusion of vegetable proteins, such as soybean meal, and supplementing the diet with industrial amino acids like lysine, methionine, and threonine, it is possible to optimize the amino acid balance, decrease the digestive load, and promote more efficient nutrient utilization. Consequently, this strategy can help reduce heat stress, improve feed efficiency, and enhance animal welfare (7, 8).

Integrating these advanced nutritional practices with environmental adjustments creates more favorable conditions for quail productivity, especially in challenging climates, and offers a sustainable and economically viable solution for poultry production in hot regions. The synergy between nutrition and environmental management presents promising benefits for the poultry industry, improving both animal welfare and sustainable production.

To date, studies in this area have examined factors such as temperature and nutrition independently, without considering their interactions. While some studies highlight the direct impact of temperature on quail performance (4, 9), others focus on the influence of different crude protein levels, with significant variations in recommended levels (8, 10). This fragmented approach highlights a gap in understanding the combined effect of temperature and nutrition on quail performance. Therefore, it is essential to investigate these interactions in an integrated way, to develop nutritional strategies that better meet the needs of quails under heat stress.

This study aims to address this gap by evaluating whether reducing crude protein in the diet, with or without amino acid supplementation, can mitigate the effects of heat stress in meat quails. The central hypothesis is that, under heat stress conditions, amino acid supplementation in low-protein diets will maintain the productive performance and carcass traits of quails while reducing metabolic heat. Thus, this research is expected to provide valuable insights for more effective, economically advantageous, and sustainable nutritional guidelines for quail production in hot climates.

## 2 Methods

### 2.1 Animals, management and experimental design

All animal use practices in this study were approved by the Institutional Animal Care and Ethics Commission for Animal Use at the Biotechnological Centre of the Federal University of Paraiba (CEUA/CBIOTEC/UFPB).

The experiment was conducted in climatic chambers located at the Poultry Nutrition Research Sector (SPNA) of the Center for Human, Social, and Agrarian Sciences (CCHSA), Campus III, Federal University of Paraiba (UFPB), Bananeiras, Paraiba, Brazil (06°45′ S, 35°38′ W, altitude 520 m). A total of 324 male European quails (*Coturnix coturnix*), sexed and with an average initial weight of 121.48 g ± 3.1 g, were evaluated from 21 to 42 days of age, over a 21-day period. The experimental design was completely randomized, comprising nine treatments (nutritional plans) with six replicates and six birds per experimental unit.

The quails were housed in galvanized wire battery cages, with a total of 54 cages measuring 67 × 37 × 20 cm (length x width x height; 413 cm^2^ per bird). Each cage was equipped with trough feeders and nipple drinkers. A photoperiod of 20 h of light and 4 h of darkness was maintained throughout the experiment.

The air temperature (TA) within the chamber was consistently maintained at ±30°C to induce heat stress, and was achieved through a set of six fluorescent heating lamps, each with a power rating of 200 watts. Temperature and humidity levels in the room were monitored daily by using a set of dry and wet bulb thermometers (Inconterm - Model: 5203.03.00; range: −10°C to +50°C; accuracy: ± 1°C) and a black globe thermometer, which consisted of a black-painted plastic sphere (0.15 m in diameter) containing a thermometer (range: −10°C to +60°C; accuracy: ± 1°C). This apparatus was positioned centrally in the room at the height of the animals’ center of mass. The recorded temperature and humidity values were subsequently converted into the Black Globe Humidity Index (BGHI) following the equation proposed by Buffington et al. (11). During the experimental period, the indoor air temperature averaged 30°C, with relative humidity at 76.42%. The BGHI was recorded at 82.19, confirming thermal stress conditions for the birds.

### 2.2 Experimental diets

**Figure 1:**
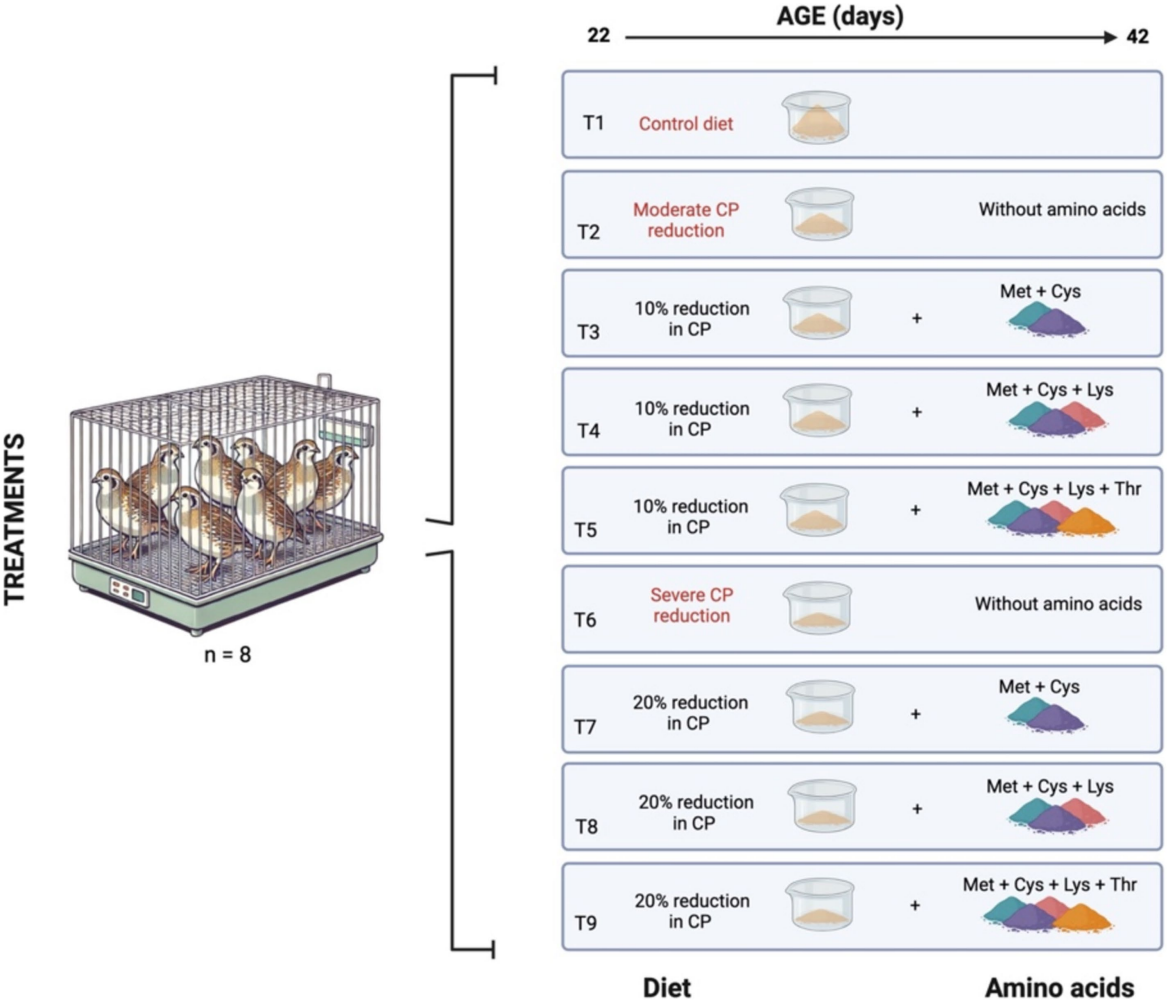
Illustration of the experimental diets fed to European quails (*Coturnix coturnix*) during the experimental period from 22 to 42 days of age (Created in https://BioRender.com).

The chemical composition of the ingredients (Table 1) used in the formulation of the experimental diets was determined through Near Infrared Spectroscopy (NIRS). Based on this analysis, isoenergetic diets (Table 2) were formulated to provide 12.76 MJ of metabolizable energy (ME) per kilogram. The control diet (T1) contained 220.00 g/kg of crude protein, fulfilling 100% of the recommended nutritional requirements (12). The other treatments varied in crude protein (CP) reduction levels and specific amino acid corrections (AC), with a focus on correcting the three first limiting amino acids for birds fed corn and soybean meal-based diets: methionine (Met), lysine (Lys), and threonine (Thr). These amino acids were corrected through the supplementation of industrial-grade methionine, lysine, and threonine, as detailed below:

- T2 diet: Moderate CP reduction (10% reduction in CP) without AC.
- T3 diet: Moderate CP reduction with correction of methionine (Met) and cystine (Cys) (10% reduction in CP + Met + Cys).
- T4 diet: Moderate CP reduction with Met + Cys and lysine (Lys) correction (10% reduction in CP + Met + Cys + Lys).
- T5 diet: Moderate CP reduction with Met + Cys, Lys, and threonine (Thr) correction (10% reduction in CP + Met + Cys + Lys + Thr).
- T6 diet: Severe CP reduction (20% reduction in CP) without AC.
- T7 diet: Severe CP reduction with Met + Cys correction (20% reduction in CP + Met + Cys).
- T8 diet: Severe CP reduction with Met + Cys and Lys correction (20% reduction in CP + Met + Cys + Lys).
- T9 diet: Severe CP reduction with Met + Cys, Lys, and Thr correction (20% reduction in CP + Met + Cys + Lys + Thr) (Figure 1).

**Table 1:** Composition determined by chemical analysis of the ingredients used in the formulation of the experimental diets.

Description	CP (g/kg)	ME (MJ/kg)	Met+Cys. dig (g/kg)	Lys dig. (g/kg)	Thr dig. (g/kg)	Calcium (g/kg)	Phosphorus available (g/kg)	Sodium (g/kg)	Chlorine (g/kg)
Corn. 7.88%	8.4	13.7578	0.33	0.21	0.26	0.03	0.24	0.00	0.06
Soybean meal. 45%	46.5	10.0651	1.11	2.50	1.55	0.30	0.62	0.03	0.05
Phosphate dicalcium	0.0	0	0.00	0.00	0.00	24.50	18.20	0.00	0.00
Limestone	0.0	0	0.00	0.00	0.00	38.00	0.00	0.00	0.00
Soybean oil	0.0	38.3636	0.00	0.00	0.00	0.00	0.00	0.00	0.00
Salt	0.0	0	0.00	0.00	0.00	0.00	0.00	39.20	60.80
DL-Methionine. 99%	58.1	19.4142	99.35	0.00	0.00	0.00	0.00	0.00	0.00
L-Lsina ● HCL. 99%	94.4	17.011	0.00	77.79	0.00	0.00	0.00	0.00	19.40
L-Threonine. 99%	73.0	14.9092	0.00	0.00	99.28	0.00	0.00	0.00	0.00
Glutamic acid 20%	60.0	14.3775	0.00	0.00	0.00	0.00	0.00	0.00	0.00
Choline chloride; 60%	0.0	0	0.00	0.00	0.00	0.00	0.00	0.00	12.70

**Table 2:** Ingredients of diets and values calculated of the nutritive composition of diets for meat quail from 22 to 42 days of age.

Treatments									
Ingredients, g/kg	Control diet	Moderate CP reduction, without AC	Moderate CP reduction plus Met + Cys	Moderate CP reduction plus Met + Cys and Lys	Moderate CP reduction plus Met + Cys. Lys and Thr	Severe CP reduction, without AC	Severe CP reduction plus Met+ Cys	Severe CP reduction plus Met + Cys and Lys	Severe CP reduction plus Met + Cys, Lys and Thr
Corn, 7.88%	617.00	656.30	654.80	654.60	654.80	692.80	691.00	691.40	691.70
Soybean meal, 45%	300.10	305.90	303.40	295.40	295.30	251.90	248.80	245.40	242.00
Phosphate dicalcium	10.00	9.80	9.90	9.90	9.90	10.20	10.20	10.20	10.30
Limestone	8.50	8.50	8.50	8.50	8.50	8.70	8.70	8.70	8.70
Soybean oil	9.10	9.10	9.10	9.10	9.10	9.10	9.10	9.10	9.10
Salt	3.30	3.20	3.30	3.30	3.30	3.30	3.30	3.30	3.30
DL-Methionine, 99%	2.30	0.00	2.20	2.30	2.30	0.00	2.70	2.70	2.80
L-Lsina ● HCL, 99%	0.00	0.00	0.00	0.00	0.00	0.00	0.00	1.60	1.70
L-Threonine, 99%	1.10	0.00	0.00	0.00	1.10	0.00	0.00	0.00	1.90
Glutamic acid 20%	45.50	0.00	0.00	6.50	5.10	0.00	0.00	0.00	0.00
Coline Chloride, 60%	1.00	1.00	1.00	1.00	1.00	1.00	1.00	1.00	1.00
Vitamine^a^	1.00	1.00	1.00	1.00	1.00	1.00	1.00	1.00	1.00
Minerals^b^	0.70	0.70	0.70	0.70	0.70	0.70	0.70	0.70	0.70
Antioxidant^c^	0.10	0.10	0.10	0.10	0.10	0.10	0.10	0.10	0.10
Anticoccidial^d^	0.20	0.20	0.20	0.20	0.20	0.20	0.20	0.20	0.20
Promotor growth^e^	0.10	0.10	0.10	0.10	0.10	0.10	0.10	0.10	0.10
Inert^f^	0.00	4.30	6.00	7.50	7.70	21.10	23.30	24.60	25.50
Total	1000.00	1000.00	1000.00	1000.00	1000.00	1000.00	1000.00	1000.00	1000.00

Nutrient composition									
CP (g/kg)	220.00	198.00	198.00	198.00	198.00	176.00	176.00	176.00	176.00
ME (MJ/kg)	12.76	12.76	12.76	12.76	12.76	12.76	12.76	12.76	12.76
Met+Cys. dig (g/kg)	8.00	5.90	8.00	8.00	8.00	5.40	8.00	8.00	8.00
Lys dig. (g/kg)	10.20	10.50	10.40	10.20	10.20	9.20	9.10	10.20	10.20
Thr dig. (g/kg)	7.80	7.00	7.00	6.80	7.80	6.20	6.20	6.10	7.80
Calcium (g/kg)	7.00	7.00	7.00	7.00	7.00	7.00	7.00	7.00	7.00
Phosphorus available (g/kg)	2.70	2.70	2.70	2.70	2.70	2.70	2.70	2.70	2.70
Sodium (g/kg)	1.50	1.50	1.50	1.50	1.50	1.50	1.50	1.50	1.50
Chlorine (g/kg)	2.50	2.50	2.50	2.50	2.50	2.50	2.50	2.80	2.90
Potassium (g/kg)	8.30	8.60	8.50	8.40	8.40	7.50	7.50	7.40	7.30

^a^Vitamin premix per kilogram of diet: Vitamin A, 10.000.000 U.I.; vitamin D3, 2.500.000 U.I.; vitamin E, 6.000 U.I.; vitamin K, 1.600 mg; vitamin B12, 11.000; Niacin, 25.000 mg; folic acid, 400 mg; pantothenic acid, 10.000 mg. ^b^Mineral premix provided per kilogram of diet: Mn (from MnSO4·H2O), 65 mg; Zn (from ZnO), 100 mg; Fe (from FeSO4·7H2O), 100 mg; Cu (from CuSO4·5H2O), 16 mg; I [from Ca (IO3)2·H2O], 1.5 mg; Se, 0.30 mg. ^c^Antioxidant = BHT, 10 g; Vehicle quantity sufficient to 1,000 g. ^d^Anticoccidial = Coban. ^e^Promotor growth of gram-positive = enradin = 100 g/ton. ^f^Washed sand.

Water and feed were provided *ad libitum.* Any feed residues on the ground were collected daily and added to the leftovers from the feeders.

### 2.3 Performance and carcass assessment

The quails were individually weighed on days 22 and 42 to determine live weight (g) and weight gain (g/day). Feed intake (g/day) and feed conversion efficiency (g/g) were calculated based on the difference between the feed provided and the leftovers in the feeders and buckets, and by relating feed intake to weight gain, respectively.

Following a 12-h fasting period, two quails per experimental unit were selected based on their mean weight (±10%) and were euthanized by using the cervical dislocation method (13, 14). The birds were subsequently plucked and eviscerated to obtain the absolute (g) and relative (%) weights of the carcass, prime cuts (breast and leg), and edible organs (heart, liver, and gizzard). The relative weight was calculated by using the formula: organ weight/bird weight × 100 (8).

### 2.4 Statistical analysis

The normality of the data was verified using skewness and kurtosis coefficients. Analyses were conducted using the least-squares method to assess the effect of the nutritional plans on performance and carcass variables. The statistical model used was an analysis of variance (ANOVA) with the following structure:

$Y_{ij}=\mu +T_{i}+\epsilon_{ij}$

where *Y_ij_* represents the observed value of the dependent variable for the *j*-th experimental unit within the *i*-th treatment, *μ* is the overall mean, *T_i_* is the fixed effect of the *i*-th treatment, and *ϵ_ij_* is the random error term, assumed to be normally distributed (*N* (0, *σ^2^*)). The analyses were performed using SAS version 9.3 (SAS Inst. Inc., Cary, NC, USA). Treatment means were compared using the Student–Newman–Keuls (SNK) test, with a significance level set at *p* < 0.05 (15).

## 3 Results

### 3.1 Performance

Reducing crude protein in the diet, with or without amino acid supplementation, did not significantly affect (*p* > 0.05) final weight and feed intake in male European quails aged 22 to 42 days. However, significant effects (*p* < 0.05) were observed on weight gain and feed conversion. Quails fed the T4 diet (moderate protein reduction with Met + Cys and Lys) and the T7 diet (severe reduction with Met + Cys) demonstrated superior performance in these parameters, as detailed in Table 3.

**Table 3:** Final weight, weight gain, feed intake, and feed conversion of meat quails at 42 days of age receiving protein-reduced diets in a high-temperature environment.

Treatments	Final weight (g bird^−1^)	Feed intake (g bird^−1^)	Weight gain (g bird^−1^)	Feed conversion (g g^−1^)
Control diet	209.58	412.02	86.88 c	4.77 b
Moderate CP reduction, without AC	209.10	427.08	91.73 b	4.71 b
Moderate CP reduction plus Met + Cys	218.36	429.50	89.89 b	4.85 b
Moderate CP reduction plus Met + Cys and Lys	217.65	420.89	97.78 a	4.37 a
Moderate CP reduction plus Met + Cys. Lys and Thr	214.18	425.08	92.76 b	4.62 b
Severe CP reduction, without AC	211.76	432.91	89.18 b	4.88 b
Severe CP reduction plus Met+ Cys	216.11	415.44	97.50 a	4.28 a
Severe CP reduction plus Met + Cys and Lys	205.30	432.66	86.27 c	5.10 c
Severe CP reduction plus Met + Cys, Lys and Thr	215.51	432.36	92.26 b	4.75 b
S.E.M.	0.38	3.8	7.6	0.12
*p* value	0.451	0.210	<0.040	<0.050
CV (%)	4.93	6.85	7.35	8.53

Means in the same column followed by different letters are different from each other by the SNK test (*p* < 0.05).

### 3.2 Carcass traits

The dietary treatments did not significantly influence (*p* > 0.05) carcass weight, carcass yield, breast weight, or leg weight of quails at 42 days of age (Table 4).

**Table 4:** Weight (g) and yield (%) of carcass, breast, and legs of meat quails at 42 days of age receiving protein-reduced diets in a high-temperature environment.

Treatments	Carcass		Breast		Legs	
	(g)	(%)	(g)	(%)	(g)	(%)
Control diet	149.33	75.26	52.65	35.34	32.88	22.06
Moderate CP reduction, without AC	145.76	73.35	50.52	34.64	33.25	22.84
Moderate CP reduction plus Met + Cys	154.48	73.90	55.32	35.86	34.22	22.18
Moderate CP reduction plus Met + Cys and Lys	150.19	72.67	55.02	36.78	33.91	22.62
Moderate CP reduction plus Met + Cys. Lys and Thr	144.92	71.37	52.10	36.36	31.65	21.93
Severe CP reduction, without AC	147.40	72.86	53.98	36.59	32.83	22.28
Severe CP reduction plus Met+ Cys	151.68	71.76	55.91	36.81	33.31	21.96
Severe CP reduction plus Met + Cys and Lys	146.45	75.95	53.40	35.90	32.24	22.05
Severe CP reduction plus Met + Cys, Lys and Thr	151.62	74.47	54.34	35.94	33.74	22.23
S.E.M.	0.71	0.70	0.80	0.75	0.43	0.65
*p* value	0.453	0.088	0.876	0.347	0.654	0.100
CV (%)	7.58	7.69	11.97	9.73	8.94	7.01

### 3.3 Organ weights

The reduction in crude protein, regardless of whether it met the essential amino acid requirements, did not significantly affect (*p* > 0.05) the weights of the heart and gizzard. In contrast, the diets significantly influenced (*p* < 0.001) both the absolute and relative weights of the liver (Table 5). The lowest liver yield was observed in quails fed the T7 diet (*p* < 0.001), which contained 17.6% crude protein along with corrections for methionine (Met) and lysine (Lys).

**Table 5:** Weight (g) and yield (%) of edible organs of European quails at 42 days of age receiving with protein reduction diets receiving protein-reduced diets in a high-temperature environment.

Treatments	Heart		Gizzard		Liver	
	(g)	(%)	(g)	(%)	(g)	(%)
Positive control	1.56	1.08	3.17	2.18	3.15 ab	2.15 ab
Light addition of CP	1.44	1.10	3.59	2.77	3.15 ab	2.37 ab
Moderate reduction plus Met + Cys	1.73	1.12	3.71	2.40	3.26 ab	2.11 ab
Moderate reduction plus Met + Cys and Lys	1.53	0.99	3.65	2.34	3.44 ab	2.21 ab
Moderate reduction plus Met + Cys. Lys. and Thr	1.39	0.96	3.42	2.38	3.57 ab	2.46 ab
Severe reduction	1.62	1.10	3.17	2.15	3.04 ab	2.07 ab
Severe reduction plus Met+ Cys	1.61	1.06	3.63	2.40	4.02 b	2.67 b
Severe reduction plus Met + Cys and Lys	1.35	0.92	3.44	2.35	2.53 a	1.73 a
Severe reduction plus Met + Cys. Lys. and Thr	1.43	0.94	3.32	2.19	3.38 ab	2.23 ab
S.E.M.	0.06	0.03	0.29	0.09	0.27	0.04
*p* value	0.547	0.238	0.078	0.099	<0.001	<0.001
CV (%)	22.06	25.86	16.89	23.78	28.49	30.92

Means in the same column followed by different letters are different from each other by the SNK test (*p* < 0.05).

## 4 Discussion

According to the data from the temperature-humidity index, the quails were effectively subjected to thermal stress, with values exceeding the thermoneutrality range of 21 to 26°C, as described by Soares et al. (4) and Saraiva et al. (8) for this species. It is noteworthy that the average temperature and temperature-humidity index observed in this study are representative of typical conditions in tropical environments, which often exhibit higher temperatures than those recorded in this experiment (16). This context underscores the necessity to adapt management practices to these extreme conditions in order to preserve animal welfare and optimize productivity in tropical systems.

It is widely recognized that thermal stress in tropical environments negatively impacts feed intake in birds. Under heat stress conditions, the metabolic heat generated from the digestion and metabolism of nutrients contributes to thermal discomfort, prompting birds to voluntarily reduce their feed intake as a means of minimizing internal heat production (17). This behavior represents a natural adaptation that, while aiding in maintaining thermal homeostasis, can compromise the intake of essential nutrients and, consequently, the productive performance of the birds. The reduction in feed intake under such conditions poses a significant challenge for the nutrition and management of birds in warm climates, highlighting the need for strategies that mitigate the effects of thermal stress and sustain productivity.

In this regard, the reduction of caloric increment in the diet, as seen in feeding regimens with lower protein levels, could theoretically alleviate the impact of thermal stress by decreasing endogenous heat production during digestion and metabolism (18). However, this effect was not observed in this study. The results indicated that even with reduced protein levels, there was no significant increase in voluntary feed intake. This suggests that thermal stress, induced by high environmental temperatures, was the predominant factor, overshadowing the potential benefits of dietary protein modulation in stimulating appetite and increasing feed intake in quails.

Although dietary variations did not substantially alter feed intake, the total intake remained low compared to the averages observed for this life stage under thermoneutral conditions. This finding corroborates the inverse relationship between feed intake and ambient temperature, a phenomenon documented by Barbosa et al. (19), who observed a reduction in quail feed intake at elevated temperatures of 27 and 35°C.

Thus, it is evident that the adverse effects of high temperature not only influence feed intake but also mask the potential dietary influences on this behavior. This notion is reinforced by Saraiva et al. (8), who reported increased feed intake and improvements in feed conversion rates in quails aged 22–42 days and maintained under thermoneutral conditions, contrasting with the results observed under thermal stress. The reduction in feed intake under heat conditions is a behavioral adaptation in birds to minimize internal heat production, which, in turn, compromises the intake of essential nutrients for protein synthesis.

Furthermore, Orhan et al. (20) emphasize the connection between thermal stress and reduced performance in birds, associating diminished nutrient digestibility with the physiological challenges imposed by high temperatures. As noted by Bonnet et al. (21), alterations in feed digestibility often reflect metabolic adaptations to thermal stress, such as accelerated passage of food through the digestive tract, resulting from increased water intake and modifications in intestinal morphology and enzymatic activity. The neuroendocrine system also responds to thermal stress with various physiological adaptations aimed at maintaining homeostasis. However, these thermoregulatory mechanisms need a redistribution of energetic resources that could otherwise be utilized for growth and muscle development.

The results of this study revealed significant effects on weight gain and feed conversion in quails, particularly in the groups fed with the T4 (moderate protein reduction supplemented with methionine [Met], cysteine [Cys], and lysine [Lys]) and T7 (severe protein reduction with Met and Cys) diets. These findings suggest that protein modulation in the diets, coupled with supplementation of essential amino acids, could be an effective strategy to improve the productive performance of quails, even under thermal stress.

The superior performance observed in quails fed with the T4 and T7 diets can be attributed to the proper balance of essential amino acids, such as methionine (Met) and lysine (Lys), which are crucial for protein synthesis and overall bird health. Methionine, in particular, is often a limiting amino acid in corn and soybean meal-based diets, which are commonly used in Brazil (22). In addition to its crucial role in muscle growth and tissue formation, methionine is also essential for feather synthesis, a process that requires a large quantity of amino acids (22). The inclusion of these limiting amino acids in the diets, such as Met + Cys and Lys, may help mitigate the negative effects of thermal stress, which typically results in reduced feed intake and, consequently, reduced intake of essential nutrients.

Moreover, the moderate and severe protein reductions in the diets, combined with essential amino acid supplementation, may have favored greater feed efficiency, enabling the quails to utilize nutrients more effectively. This factor is particularly important under high temperature conditions, where thermal stress reduces feed intake, impairing weight gain and feed conversion. The reformulation of the diets, with a balanced ratio of essential amino acids, likely provided additional support to performance, even with reduced crude protein, helping to maintain bird productivity in challenging environments.

These results support the hypothesis that adjustments in dietary formulations, considering the quantity and quality of the nutrients, can be a viable strategy to optimize quail performance, especially under adverse conditions such as thermal stress. Thus, the T4 and T7 diets not only showed promising results, but also provided valuable insights for formulating diets in poultry production systems, aiming for economic efficiency and animal welfare, in a context of growing concerns with sustainability and environmental management in poultry farming.

Additionally, research by Baziz et al. (23) suggests that elevated temperatures may negatively affect the musculature of quails, particularly the breast and leg muscles. This effect would be caused by disruptions in the use of energy substrates, such as glucose and fatty acids, and alterations in protein metabolism, which could explain the observed differences in muscle protein gain and muscle development in birds under thermal stress.

In the present study, the absence of significant impact of dietary treatments on carcass weight and yield, as well as on the breast and leg weights of quails at 42 days of age, suggests that the primary muscle development was preserved, even in diets with reduced crude protein without amino acid corrections. However, the inclusion of essential amino acids in reduced-protein diets may have played an important role in mitigating potential adverse effects of thermal stress on muscle metabolism, optimizing nutrient utilization, and enhancing other performance parameters, such as weight gain and feed conversion (24).

These observations reinforce the idea that, although carcass yield and main cuts can be maintained in protein-reduced diets, amino acid supplementation may act as a metabolic protective factor against thermal stress, improving feed efficiency and maximizing muscle performance under high-temperature conditions.

In general, the organ weights of quails subjected to different dietary treatments were lower than the values typically observed under thermoneutral conditions, which may indicate an adaptation to heat stress. This adaptation could be a reflection of a lower metabolic rate in birds under high temperatures, in contrast to the higher metabolic activity that occurs under thermoneutrality. Santos et al. (25) also reported that elevated temperatures may limit organ development, restricting the generation of metabolic heat and functioning as an adaptive mechanism in birds exposed to thermal discomfort.

The results demonstrate that the reduction of crude protein in the diets, with or without supplementation of essential amino acids, did not significantly affect the absolute and relative weights of the heart and gizzard, corroborating findings by Oliveira (26), who also did not observe variations in these organs with different protein levels in quails. However, liver weight was significantly affected by the diets. Quails fed with the T7 diet, which contained 17.6% crude protein, supplemented with methionine (Met) and lysine (Lys), showed lower liver weight. This suggests that an adequate amino acid balance may help reduce metabolic overload in the liver.

Hepatic hypertrophy is generally associated with an imbalance of amino acids, leading the liver to increase its metabolic activity to compensate for deficiencies or excesses. This adaptive response can result in increased liver weight, as observed in less balanced diets (27). Thus, the better formulation of the T7 diet, by avoiding overloads in the liver, suggests more efficient liver function, which is essential for the healthy development of quails.

The results of this study reinforce the feasibility of reducing crude protein in quail diets, with both economic and environmental benefits. In Brazil, where soybean meal is an expensive input in poultry farming, the inclusion of limiting amino acids such as methionine, lysine, and threonine, available at commercial levels, not only reduces costs but also maintains productive efficiency. This approach also contributes to reducing nitrogen emissions, promoting greater sustainability in the sector.

In summary, this research highlights the complex relationship between dietary protein levels, amino acid supplementation, and the physiological responses of quails under thermal stress. The results emphasize the importance of innovative nutritional strategies that prioritize productive efficiency and environmental sustainability, contributing to the advancement of poultry science and industrial practices. Future research should continue to investigate the optimal balance between dietary components and environmental management techniques, aiming to improve bird welfare and productivity under challenging climatic conditions.

## 5 Conclusion

This study shows that, in hot environments, reducing the crude protein content in feed for heat-stressed meat quails from 22 to 17.6% is feasible when supplemented with methionine, achieving digestible Met + Cys levels of 0.800% during the growth phase (22–42 days). The results suggest that tailored nutritional strategies, particularly under heat stress conditions, may optimize zootechnical performance in lower-protein diets, reduce costs, and provide environmental benefits, such as decreasing nitrogen excretion, through appropriate amino acid supplementation. It is recommended that future research continue to explore the interactions between diet, thermal stress, and quail performance, including the analysis of different combinations of amino acids and their respective influences on the health and productivity of birds under varying temperature conditions.

## Data Availability

The raw data supporting the conclusions of this article will be made available by the authors, without undue reservation.
